# Understanding the polypharmacological anticancer effects of Xiao Chai Hu Tang via a computational pharmacological model

**DOI:** 10.3892/etm.2014.1660

**Published:** 2014-04-02

**Authors:** CHUN-SONG ZHENG, YIN-SHENG WU, HONG-JUAN BAO, XIAO-JIE XU, XING-QIANG CHEN, HONG-ZHI YE, GUANG-WEN WU, HUI-FENG XU, XI-HAI LI, JIA-SHOU CHEN, XIAN-XIANG LIU

**Affiliations:** 1 Academy of Integrative Medicine, Fujian University of Traditional Chinese Medicine, Fuzhou, Fujian 350122, P.R. China; 2 Department of Pharmacy, Xiamen Medical College, Xiamen, Fujian 361008, P.R. China; 3 College of Chemistry and Molecular Engineering, Peking University, Beijing 100871, P.R. China

**Keywords:** Xiao Chai Hu Tang, polypharmacology, cancer, computational pharmacology

## Abstract

Xiao Chai Hu Tang (XCHT), a traditional herbal formula, is widely administered as a cancer treatment. However, the underlying molecular mechanisms of its anticancer effects are not fully understood. In the present study, a computational pharmacological model that combined chemical space mapping, molecular docking and network analysis was employed to predict which chemical compounds in XCHT are potential inhibitors of cancer-associated targets, and to establish a compound-target (C-T) network and compound-compound (C-C) association network. The identified compounds from XCHT demonstrated diversity in chemical space. Furthermore, they occupied regions of chemical space that were the same, or close to, those occupied by drug or drug-like compounds that are associated with cancer, according to the Therapeutic Targets Database. The analysis of the molecular docking and the C-T network demonstrated that the potential inhibitors possessed the properties of promiscuous drugs and combination therapies. The C-C network was classified into four clusters and the different clusters contained various multi-compound combinations that acted on different targets. The study indicated that XCHT has a polypharmacological role in treating cancer and the potential inhibitory components of XCHT require further investigation as potential therapeutic strategies for cancer patients.

## Introduction

Cancer is a leading cause of mortality worldwide, accounting for 7.6 million fatalities (around 13% of total fatalities) in 2008. Cancer-associated fatalities worldwide are projected to continue to rise to >13.1 million by 2030 (1,2). To date, the commonly adopted approaches to cancer treatment are surgery, radiotherapy and chemotherapy. However, there are increasing limitations resulting from poor prognosis and the instance of negative side-effects (3,4). Furthermore, carcinogenesis involves multiple genetic and epigenetic changes; therefore, a single chemopreventive agent may not be sufficient to prevent these events (5). In addition, it has been observed that certain effective therapeutic agents act on multiple targets rather than one specific disease-associated target (6). Therefore, the use of a combination of agents that have multi-target effects may be an improved treatment strategy for cancer.

There is increasing evidence that Chinese herbal medicines are frequently administered to counteract the side-effects of chemotherapy and radiotherapy in patients who are being treated for cancer (5,7), and they have been adopted as adjuvants for cancer therapy in the USA (8). Furthermore, natural products have been indicated to be more promising candidates for cancer treatments than purely synthetic compounds (9). The earliest Chinese medicine book, *Huangdi neijing*, referred to symptoms that were comparable to those of cancer >2,000 years ago (10). Herbal medicines containing numerous constituents demonstrate variable pharmacological activities; thus, multi-herb therapy may be an effective, conventional and complementary medical approach for cancer prevention and control (11). Therefore, understanding the molecular mechanisms of Chinese herbal medicines may aid the modernization of herbal remedies and the discovery of novel cancer treatments.

Xiao Chai Hu Tang (XCHT), a traditional herbal formula from the medical treatise Shang Han Lun that was developed by Zhang in the Eastern Han Dynasty, has been administered for the treatment of cancer in China (10,11). Data from recent studies have demonstrated that XCHT treats cancer by enhancing immune regulation, anti-angiogenesis and the apoptosis of tumor cells (11–13). However, the underlying molecular mechanisms of its anticancer effects are poorly understood. Therefore, the aim of the present study was to elucidate the multi-target effects of the compounds in XCHT, based on an established computational pharmacological model (14,15). The information may aid the development of a combination of agents for the treatment of cancer.

## Materials and methods

### Collection and chemical space mapping of the compounds in XCHT

XCHT consists of seven medicinal herbs, namely *Radix Bupleuri, Scutellaria baicalensis, Panax ginseng, Zizyphi Fructus, Pinellia ternata, Zingiber officinale and Radix Glycyrrhizae*. A total of 434 non-duplicated compounds present in these herbs were identified in the Chinese Herbal Drug Database and the Handbook of the Constituents in Chinese Herb Original Plants (16,17). The two-dimensional (2D) structures of the compounds were drawn using ISIS Draw version 2.5 (MDL Information Systems, Inc., San Leandro, CA, USA), transformed into 3D-molecule models and optimized using Discovery Studio 2.0 (DS 2.0; Accelrys, Inc., San Diego, CA, USA) with a Merck molecular force field. Using the quantitative structure-activity relationship module of DS 2.0 (Accelrys, Inc.), 150 diversity descriptors of the compounds, including 1D-, 2D- and 3D-molecular descriptors (14), were calculated. Principal component analysis (PCA) was performed to map the chemical space distribution of the compounds.

### Molecular docking

The crystal structures of the protein-ligand complexes for the 29 cancer-associated targets were used for the docking calculations that were performed using the LigandFit module within the DS 2.0 software (18,19). They were downloaded from the Research Collaboratory for Structural Bioinformatics (RCSB) Protein Data Bank (PDB; http://www.rcsb.org/pdb/home/home.do; Table I). For each crystal structure, the crystallographic water molecules were removed, the missing hydrogen atoms were added and the inhibitor from the crystal structure was used to define the active site. The 434 compounds identified as being present in XCHT were docked into the protein models and the interactions between the compounds and proteins were evaluated using a DockScore (20). The 434 docked structures were sorted according to their DockScore, in readiness for network construction being undertaken.

### Network construction and analysis

Cytoscape 2.8.3 (The Cytoscape Consortium, San Diego, CA, USA) was used to construct the subsequent networks (21,22). In order to construct the compound-target (C-T) network, a compound node and a target node were linked if the DockScore of the compound and the target was in the top 13 (top 3%) of all the compounds (15). If two compounds shared ≥1 target, they were linked to create a compound-compound (C-C) association network. The data were analyzed using Cytoscape plugins (The Cytoscape Consortium).

## Results

### Chemical space distribution of the compounds in XCHT

**Figure 1:**
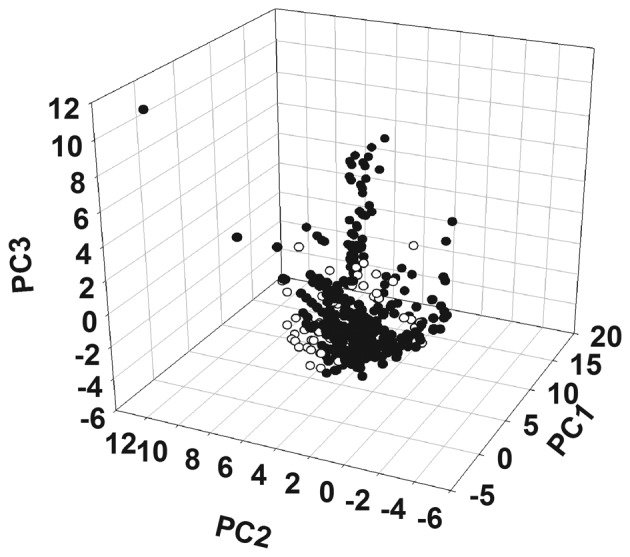
Map demonstrating the chemical space of the compounds in Xiao Chai Hu Tang (black circles) and the known inhibitors (white circles) that are associated with cancer targets, obtained from the Therapeutic Targets Database. PC1, first principal component; PC2, second principal component; PC3, third principal component.

The chemical space of XCHT was mapped based on the PCA and the results of the mapping are shown in Fig. 1 and Table II. The distribution of XCHT in the chemical space ranges from dense to loose. Table II shows that the means of the molecular weight, the number of hydrogen-bond donors, the number of hydrogen-bond acceptors and AlogP were 376.26, 3.26, 5.94 and 2.71, respectively. According to Lipinski’s rule of five (23), the majority of the compounds in XCHT exhibited drug-like properties. To further demonstrate the drug-like properties of the compounds in XCHT, the map of chemical space of the known inhibitors that are associated with cancer targets, according to the Therapeutic Targets Database (TTD) was also constructed (Fig. 1) (18). The majority of the known inhibitors and the compounds present in XCHT were observed to have clustered at the bottom back corner of the chemical space.

### Identification of the multi-target compounds in XCHT that are associated with cancer therapy

**Figure 2:**
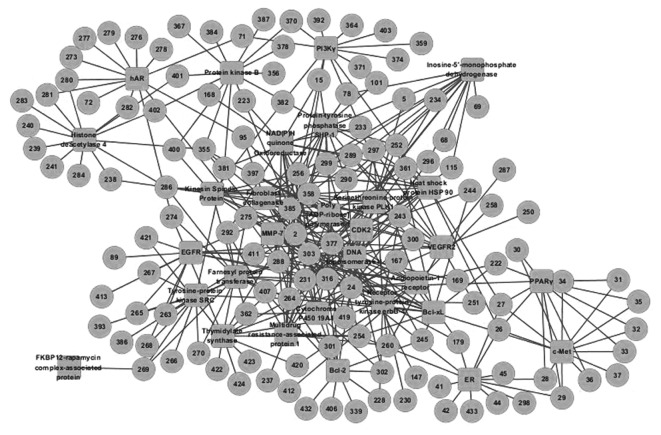
XCHT compound-target network. The rectangles and circles represent the target proteins that are associated with cancer and the XCHT compounds, respectively. The numbers indicate the index numbers of the compounds. XCHT, Xiao Chai Hu Tang.

The docking results indicated that >80% of the potential inhibitors interacted with fewer than three targets; however, a small number of compounds interacted with a large number of targets, up to a maximum of 18. Based on the screened compounds and their corresponding targets, the C-T network was generated (Fig. 2). The network parameters and key compounds of the C-T network are listed in Tables III and IV, respectively. The results demonstrate that certain predicted inhibitors present in XCHT possess the properties of promiscuous drugs and combination therapies.

### Multi-compound combination therapy of XCHT for cancer

**Figure 3:**
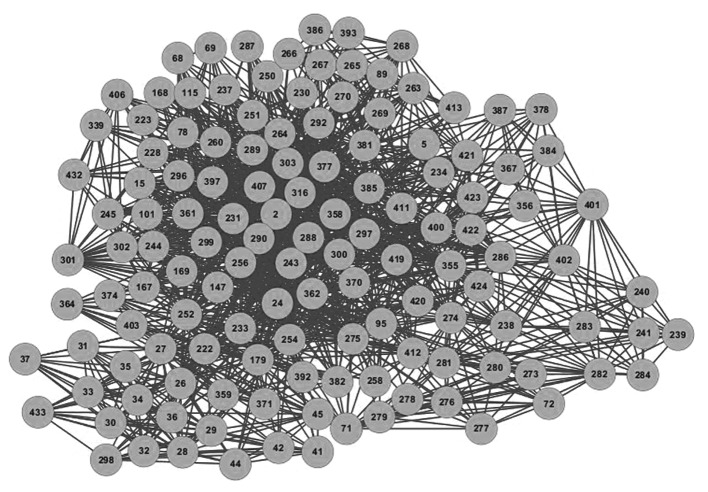
Xiao Chai Hu Tang compound-compound association network. The numbers indicate the index numbers of the compounds.

**Figure 4:**
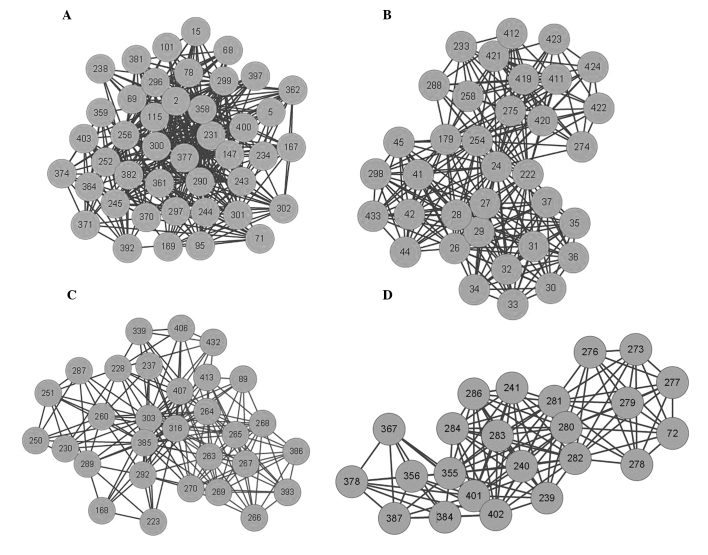
Subnetworks made up of highly interconnected regions. The circles represent compounds present in Xiao Chai Hu Tang. (A) cluster 1, (B) cluster 2, (C) cluster 3 and (D) cluster 4.

To understand the association between the potential inhibitors, the C-C network was constructed (Fig. 3). The network parameters of the C-C network are listed in Table III. A Cytoscape GLay plugin, which automatically transforms the input network into a simplified model (22), was used to identify four separate clusters within the C-C network (Fig. 4; Table V) and to demonstrate the different multi-compound and multi-herb combinations within XCHT. Furthermore, the cluster results indicate that different multi-compound combinations were able to act on different targets.

## Discussion

Traditional Chinese medicine (TCM) has been widely adopted for cancer care in China and other Asian countries, and is increasingly used as a complementary therapy by Western cancer patients (24). However, there are numerous questions regarding TCM and a lack of modern scientific language for describing it. In the present study, a computational pharmacological model was constructed to investigate the molecular characteristics and cancer therapeutic mode of a common TCM, XCHT, as an example to demonstrate the potential application of the model.

Notably, a significant proportion of the compounds in XCHT were clustered in a specific region of chemical space. According to the theory of chemical space (25), these compounds possessed similar functions, which indicates that the compounds present in the herbal components of XCHT are compatible with each other. It may provide the scientific basis for the composition of a formulation from a combination of these herbs or compounds. Furthermore, these compounds occupy a chemical space that is the same as or close to that occupied by other known inhibitors that exhibit the anticarcinogenic drug-like properties of XCHT. A minor proportion of the compounds within XCHT were dispersed and at intervals from other compounds, which corresponds with the various functions of XCHT that have been observed in a clinical setting (26,27). These may provide the foundation for the screening of suitable active compounds from XCHT that may be used as therapeutic agents against cancer.

A C-T network was constructed, based on molecular docking, to elucidate the therapeutic efficacy of XCHT against cancer. The results demonstrated that the maximum number of targets that a single compound was able to act on was 18, which indicated that XCHT is a broad-spectrum formula that has the ability to inhibit numerous significant target proteins. Furthermore, the C-T network (Fig. 2) indicates that the multiple potentially active compounds in XCHT are able to interact with various cancer-associated targets and that limited individual compounds, termed promiscuous drugs, are able to interact with multiple targets. Table III demonstrates the biological activities of certain compounds associated with the herbal components of XCHT that have been reported in the literature (28–33). Due to the molecular complexity of cancer, multi-targeted therapies are becoming increasingly important as, in the long-term, they maximize the therapeutic effect and overcome the mechanisms of resistance (34,35). Thus, the properties of multi-targeted therapies that have been identified in XCHT may be a key explanation for why XCHT is effective as an anticancer treatment.

To further investigate the therapeutic mode of XCHT against cancer, a C-C network was constructed and the potentially active compounds were clustered. As shown in Fig. 4, there were four clusters containing different compounds that may serve to provide different combination therapies and pharmacological functions. In addition, the botanical sources of each cluster were identified to be different. This may explain why XCHT has utility in the treatment of pancreatic, stomach, liver and lung cancer (26,27); furthermore, each cluster interacted with certain common targets. Generally, the range of the network clustering coefficient is between 0 and 1; the greater the coefficient, the higher the clustering property. The clustering coefficient of the C-C network in the present study was 0.829, which indicates that this particular network has a high clustering property. The compounds in high clustering have the similar activity, so they may have the synergistic combined effects. This indicated that XCHT may have a synergistic combined effect. In addition, the botanical sources of the compounds in cluster 3 include all the ingredients of XCHT (Table V), which further confirms the rationality of the specific herbal combination used in XCHT. In addition, it has been identified that XCHT in holistic combinations possesses various pharmacological properties due to the different components attacking various targets or different steps in the pathologic process of cancer (11–13). Therefore, the components of XCHT, which have different mechanisms of anti-cancer action, interact primarily in an additive or synergistic manner.

In conclusion, a computational pharmacological model was employed to illustrate the multi-compound, multi-target and multi-combination therapeutic mechanism of XCHT. The results provide an indication of the polypharmacological anticancer effect of XCHT and may aid the identification of novel therapeutic strategies for cancer patients based on the active compounds present in XCHT.

## Figures and Tables

**Table I:** Twenty-nine key target proteins that are associated with cancer.

Protein	PDB code
Human androgen receptor	1E3G
Epidermal growth factor receptor	1M17
Farnesyl protein transferase	1SA4
Fibroblast collagenase	2TCL
Heat shock protein 90	1UYD
Inosine-5′-monophosphate dehydrogenase	1ME9
Kinesin spindle protein	2FL6
Multidrug resistance-associated protein 1	2CBZ
NAD(P)H quinone oxidoreductase	1KBQ
Poly [ADP-ribose] polymerase-1	1UK0
Protein-tyrosine phosphatase SHP-1	1FPR
Receptor tyrosine-protein kinase erbB-4	3BBT
Serine/threonine-protein kinase PLK1 2RKU VEGFR2	3B8R
Tyrosine-protein kinase SRC	1FMK
Bcl-xL	1YSI
Bcl-2	2022
CDK2	1AQ1
Cytochrome P450 19A1	3EQM
DNA topoisomerase II	1ZXM
Angiopoietin-1 receptor	2OO8
Estrogen receptor	1UOM
c-Met	3EFJ
Histone deacetylase 4	2VQV
MMP-7	1MMR
PPARγ	1I7I
PI3Kγ	2A5U
Protein kinase B	3CQU
Thymidylate synthase	1CI7

PDB, Protein Data Bank; NAD(P)H, nicotinamide adenine dinucleotide phosphate-oxidase; VEGFR2, vascular endothelial growth factor receptor 2; CDK2, cyclin-dependent kinase 2; MMP-7, matrix metalloproteinase-7; PPAR, peroxisome proliferator-activated receptor; PI3K, phosphoinositide 3-kinase.

**Table II:** Mean, SD, minimum and maximum of the key molecular descriptors of compounds in Xiao Chai Hu Tang.

Molecular descriptor	Mean	SD	Minimum	Maximum
No. of carbon atoms	20.75	13.47	2	60
No. of nitrogen atoms	0.12	0.66	0	7
No. of oxygen atoms	5.87	6.31	0	29
Molecular weight	376.26	272.67	58.03	1271.44
No. of hydrogen acceptors	5.94	6.32	0	29
No. of hydrogen donors	3.26	3.88	0	19
AlogP	2.71	2.78	−7.58	19.52

SD, standard deviation; AlogP, logarithm of 1-octanol/water partition coefficient.

**Table III:** Network properties of the C-T and C-C networks.

Parameters	C-T network	C-C network
Network density	0.029	0.784
Network heterogeneity	1.045	0.675
Network centralization	0.084	0.494
Characteristic path length	3.687	1.986
Average no. of neighbors	4.696	22.809
Shortest path	25760 (100%)	17030 (100%)
Cluster coefficient	0	0.829

C-T, compound-target; C-C, compound-compound association.

**Table IV:** The 15 compounds the highest degree of target interaction in the compound-target network.

Index	Known interaction	Chemical name	Degree
377	Yes	Scutellarin	18
24	No	Folic acid	17
303	Yes	Vicenin-2	15
385	No	5,7,4′-Trihydroxy- 6-C-arabinoside-8- C-glucoside flavone	15
358	No	Dihydrobaicalin	13
316	No	Kaempferitrin	13
2	No	Adenosine triphosphate	13
275	Yes	Liquiritin	8
231	No	Gancaonin E	8
243	No	Glycyrrhisoflavone	7
264	No	Licorice saponin C2	6
288	No	Neoisoliquiritin	6
254	No	Isoliquiritin	6
355	Yes	Baicalein	5
252	Yes	Isolicoflavonol	5

**Table V:** Significant information associated with the categories of the subnetworks.

Cluster	Effector targets	Botanical source of the compounds
Cluster 1	VEGFR2, tyrosine-protein kinase SRC, thymidylate synthase, serine/threonine-protein kinase PLK1, receptor tyrosine-protein kinase erbB-4, protein-tyrosine phosphatase SHP-1, protein kinase B, poly [ADP-ribose] polymerase-1, PI3Kγ, NAD(P)H quinone oxidoreductase, multidrug resistance-associated protein 1, MMP-7, kinesin spindle protein, inosine-5′-monophosphate dehydrogenase, histone deacetylase 4, HSP 90, hAR, fibroblast collagenase, farnesyl protein transferase, EGFR, DNA topoisomerase II, cytochrome P450 19A1, CDK2, Bcl-xL, Bcl-2 and angiopoietin-1 receptor.	*Scutellaria baicalensis*, *Panax ginseng*, *Zingiber officinale*, *Zizyphi fructus*, *Radix glycyrrhiza*
Cluster 2	ER, Bcl-2, Bcl-xL, CDK2, cytochrome P450 19A1, EGFR, ER, farnesyl protein transferase, fibroblast collagenase, HSP 90, hAR, kinesin spindle protein, MMP-7, multidrug resistance-associated protein 1, NAD(P)H quinone oxidoreductase, PI3Kγ, poly [ADP-ribose] polymerase-1, PPARγ, protein-tyrosine phosphatase SHP-1, receptor tyrosine-protein kinase erbB-4, serine/threonine-protein kinase PLK1, thymidylate synthase and VEGFR2.	*Panax ginseng*, *Zizyphi fructus*, *Zingiber officinale*, *Scutellaria baicalensis*, *Pinellia ternata*, *Radix glycyrrhizae*
Cluster 3	VEGFR2, tyrosine-protein kinase SRC, thymidylate synthase, serine/threonine-protein kinase PLK1, receptor tyrosine-protein kinase erbB-4, protein-tyrosine phosphatase SHP-1, protein kinase B, poly [ADP-ribose] polymerase-1, PI3Kγ, NAD(P)H quinone oxidoreductase, multidrug resistance-associated protein 1, MMP-7, kinesin spindle protein, HSP 90, fibroblast collagenase, EGFR, farnesyl protein transferase, DNA topoisomerase II, cytochrome P450 19A1, CDK2, Bcl-xL, Bcl-2, angiopoietin-1 receptor and tyrosine-protein kinase SRC.	*Radix bupleuri*, *Panax ginseng*, *Zizyphi fructus*, *Zingiber officinale*, *Scutellaria baicalensis*, *Pinellia ternata*, *Radix glycyrrhizae*
Cluster 4	protein kinase B, farnesyl protein transferase, fibroblast collagenase, hAR, histone deacetylase 4, kinesin spindle protein, MMP-7, poly [ADP-ribose] polymerase-1 and serine/threonine-protein kinase PLK1.	*Panax ginseng*, *Zizyphi fructus*, *Scutellaria baicalensis*, *Radix glycyrrhizae*

VEGFR2, vascular endothelial growth factor receptor 2; PLK1, polo-like kinase 1; PI3Kγ, phosphoinositide 3-kinase-γ; NAD(P)H, nicotinamide adenine dinucleotide phosphate-oxidase; MMP-7, matrix metalloproteinase-7; HSP 90, heat shock protein 90; hAR, human androgen receptor; PPARγ, peroxisome proliferator-activated receptor-γ; EGFR, epidermal growth factor receptor; CDK2, cyclin-dependent kinase 2; ER, estrogen receptor.

## Abbreviations

XCHT: Xiao Chai Hu Tang
TTD: Therapeutic Targets Database
hAR: human androgen receptor
ER: estrogen receptor
VEGFR2: vascular endothelial growth factor receptor 2
MMP: matrix metalloproteinase
PPARγ: peroxisome proliferator-activated receptor-γ
PI3Kγ: phosphoinositide 3-kinase-γ
CDK2: cyclin-dependent protein kinase 2
C-T network: compound-target network
C-C network: compound-compound association network
TCM: tradition Chinese medicine
